# Cuprizone-Dependent De/Remyelination Responses and Functional Correlates in Mouse Strains Adopted to Model Relapsing, Chronic and Progressive Experimental Autoimmune Encephalomyelitis

**DOI:** 10.1007/s12640-021-00331-3

**Published:** 2021-01-21

**Authors:** Daniela Buonvicino, Giuseppe Ranieri, Alberto Chiarugi

**Affiliations:** Department of Health Sciences, Section of Clinical Pharmacology and Oncology, University of Florence, Viale Pieraccini 6, 50139 Firenze, Italy

**Keywords:** Cuprizone, NOD mice, C57BL/6 mice, SJL mice

## Abstract

NOD mice represent a unique strain that recapitulates some aspects of progressive MS when subjected to experimental autoimmune encephalomyelitis (EAE). It is unknown, however, whether a proneness to demyelination and/or defect in remyelination contribute to disease progression in NOD mice. Answering to this question might help deciphering the molecular and cellular events underpinning disease evolution in progressive MS. Here, we compared the cuprizone-dependent demyelination and remyelination responses, as well as their functional correlates, in NOD, C57BL/6, and SJL mice typically adopted to model progressive, chronic or relapsing EAE. We report that demyelination occurred to a similar extent in the three mice strains, and that in none of them there was evidence of axonal degeneration during prolonged demyelination. Moreover, immunostaining for GFAP^+^ astrocytes, Iba1^+^ microglia, and NG2^+^ oligodendrocyte precursor cells similarly increased in the 3 mouse strains after cuprizone exposure. The mice underwent concomitant and complete remyelination 2 weeks after cuprizone withdrawal. On a functional level, NOD mice showed the earliest reduction of spontaneous motility and full recovery, but no impairment of motor skill. Conversely, C57BL/6 animals showed phasic reduction of both spontaneous motility and motor skill. Lastly, SJL mice presented the most severe neurological impairment with long-lasting reduction of spontaneous motility and motor skill. Overall, data suggest that the unique feature of EAE progression in NOD mice is not due to proneness to demyelination or intrinsic defects in myelin formation. Findings also unravel important functional differences in the response of the three mouse stains to cuprizone that can be harnessed to design and interpret future experiments.

## Introduction

Multiple sclerosis (MS) is an autoimmune disorder of the central nervous system that affects about 2.3 million people worldwide and the leading cause of disability among young individuals (Thompson et al. 2018). Although the etiology of the disorder is still obscure, the pathogenesis is in part deciphered and encompasses a complex interplay of adaptive and innate immune responses initially directed against olygodendrocyte and myelin (Kutzelnigg and Lassmann 2014). Recently, the therapeutic armamentarium available to counteract MS significantly expanded with multiple drugs able to target key molecular and cellular events responsible for the autoimmune attack to the CNS (Ontaneda et al. 2019).

The major therapeutic achievements, however, are related to the treatment of the relapsing–remitting (RR) form of MS. Conversely, with the notable exception of the recent identification of ocrelizumab (Montalban et al. 2017) and siponimod (Kappos et al. 2018), drugs able to counteract progressive MS (PMS) are still an unmet need, and their development represents a key challenge for PMS therapy. Unfortunately, the mechanisms responsible for disease progression are still not understood, and a great deal of effort is directed at elucidating the molecular events underpinning neurodegeneration and axonal loss during disease evolution. In this light, reliable animal models of PMS might help to understand the pathophysiology of progression, as well as therapeutic remedies able to suppress it. The prototypical animal model of MS is experimental autoimmune encephalomyelitis (EAE) (Bert et al. 2011). Although several mouse strains undergo EAE characterized by relapses and remissions (Skundric 2005), thereby resembling RRMS, rodent models showing consistent disease progression similar to PMS are very scarce (Lassmann and Bradl 2017; Bjelobaba et al. 2018).

In this regard, we recently characterized the EAE NOD mouse model and provided neurological, neuropathological, and immune evidence that it recapitulates key features of PMS (Buonvicino et al. 2019). In particular, we showed that, at variance with the RREAE in SJL mice, or chronic EAE in the C57BL/6 mouse strain, in EAE NOD mice slowly but inexorably progresses to a severe neurological impairment that always leads to death without signs of disease stabilization (Buonvicino et al. 2019, 2020). Importantly, the neuropathological correlate consists in widespread spinal cord demyelination that is invariably accompanied by axonal loss heralded by impairment of mitochondrial structure and function. We also reported that whereas progression of EAE in NOD mice is insensitive to immune suppression (Buonvicino et al. 2019), it can be counteracted by the bioenergetic-boosting drug dexpramipexole (Buonvicino et al. 2020). These findings, on the one hand are consistent with the hypothesis that virtual hypoxia, rather than autoimmune processes, contribute to neurodegeneration during disease progression (Trapp and Stys 2009), and on the other strengthen the reliability of EAE in NOD mice as a model of PMS.

In spite of these achievements, however, the neuropathological basis of disease progression in EAE NOD mice as well as why other mouse strains do not show the same feature of disease evolution are still unknown. In the present study, we wondered whether peculiarities in the demyelination and/or remyelination events might contribute to EAE progression in NOD mice. Accordingly, a defect in myelination signaling has been detected in PMS patients (Lassmann et al. 2012; Nicaise et al. 2017, 2019). We therefore studied demyelination and remyelination responses to the oligodendrocyte-toxic agent cuprizone in NOD mice, and compared them to those triggered by the same agent in SJL and C57BL/6 mice taken as prototypical RR- and chronic EAE models. In these three mouse strains, we also the compared the neurological correlates during cuprizone exposure and subsequent withdrawal.

## Experimental Procedures

### Animals

All animal care and experimental procedures were performed according to the European Community guidelines for animal care (European Communities Council Directive 2010/63/EU) and were approved by the Committee for Animal Care and Experimental Use of the University of Florence. Female NOD/ShiLtj, SJL and C57BL/6 mice (Charles River, Milan, Italy) were housed in a conventional unit (5–6 per cage) with free access to food and water, and maintained on a 12 h light/dark cycle at 21°C room temperature. In NOD mice glycaemia was measured every week by glucometer as reported (Buonvicino et al. 2019).

### Induction of De- and Remyelination

Five-week-old female NOD/ShiLtj, SJL and C57BL/6 mice were fed with a diet containing 0.4% cuprizone (oxalic acid bis (cyclohexylidene hydrazide); Sigma-Aldrich, Milan, Italy) mixed into their normal chow (Harlan Global Diet 2018, Harlan Laboratories, Udine, Italy) for 6 weeks. After cuprizone feeding, mice were changed to standard rodent chow and subsequent remyelination was assessed 2 weeks after the end of cuprizone feeding.The total number of mice per strain exposed to cuprizone was 20.

### Histological Analysis

Histological analysis has been performed in 10 mice per strain. The animals’ brains were collected after 6 weeks of cuprizone diet (*n* = 5 per strain) or 2 weeks after cuprizone withdrawal (*n* = 5 per strain). Brains were fixed in 4% formaldehyde in 0.1 M PBS, embedded in paraffin, and cut into 10 µm thin sections. Axonal loss and myelin loss were assessed by Bielschowsky’s silver staining and Luxol Fast Blue, respectively. For each brain, serial sections of the corpus callosus portion have been collected at a 1 mm interval and distributed onto slides for a total of 3 sections per slide. Images were acquired by using an Olympus BX40 microscope (Olympus, Milan, Italy) and a digital camera (Olympus DP50) with NIS-Elements software; sections were analyzed by a blinded operator by using ImageJ software (Buonvicino et al. 2019).

### Board Test

During cuprizone exposure and its subsequent withdrawal, spontaneous motility was evaluated by board test throughout the entire course of the experiments in 10 mice per strain. Mice were placed on the center of a 40 cm square plane one by one and allowed to move about freely. Two electric sensors, crossing the plane from midpoint to midpoint of opposite sides, thus dividing the plane into four equal quadrants, automatically signaled the movement of the animal on the surface of the plane. The total counts of spontaneous locomotion in 10 min was reported for each mouse (Buonvicino et al. 2019).

### Rota Rod

Motor skill was assessed by measuring riding times on a rota rod apparatus (rotating at speed of 30 RPM) as reported (Jones and Roberts 1968). After an adaptation period of 5 days of daily practice on the rota rod, the frequency of the test was reduced every 10 days during cuprizone exposure and its subsequent withdrawal. The longest riding time that each mouse remained on the rod out of three attempts was recorded. For comparison of the performance between the three strains, 300 s was chosen as the arbitrary maximum cutoff time. Motor skill has been evaluated in 10 mice per strain.

### Immunohistochemistry

For immunohistochemical analysis, brains were collected after 6 weeks of cuprizone diet (*n* = 4 per strain), 10 µm thin sections were blocked with 5% normal goat serum (Thermo Fisher Scientific, Waltham, MA, USA) containing 0.3% Triton X-100 (Sigma, Milan, Italy). One hour later, sections were incubated overnight at 4°C with a rabbit polyclonal anti ionized calcium-binding adapter molecule 1 (Iba1) (1:300, WAKO, Osaka, Japan) for microglia, with a mouse polyclonal antibodies against glial fibrillary acidic protein (GFAP) (1:500, Cell Signaling Technologies, Beverly, MA, USA) or with a mouse monoclonal antibody against neural/glial antigen 2 (NG2) (1:500, Millipore Merck, Darmstadt, Germany). After washings, sections were incubated with an anti-rabbit secondary antibody conjugated with AlexaFluor 546 (1:2000, Thermo Fisher Sci., Waltham, MA, USA) or with an anti-mouse secondary antibody Cy3-conjugated (1:1000, Jackson ImmunoResearch, Cambridgeshire, UK). Images were acquired under a LEICA TCS SP5 confocal laser scanning microscope (Leica Microsystems CMS GmbH, Mannheim, Germany) with a × 10 and × 20 objective. Quantification of astrocytes and microglia immunofluorescence was performed using ImageJ. Values correspond to the mean of six different microscopic fields of three different mouse brain sections.

### Statistical Analysis

Data were tested for normality with Kolmogorov–Smirnov test. Data with parametric distribution were expressed as mean ± SEM. All differences were statistically evaluated comparing different conditions using ANOVA plus Tukey’s or Dunnett’s test. Data with non parametric distribution were tested with Kruskal–Wallis test followed by Dunn’s post test and expressed with median and IQR. The alpha level was fixed at 0.05. Statistical analyses were carried out using GraphPad Prism.

## Results

### Effect of Cuprizone on Callosal Demyelination and Remyelination in NOD, C57BL/6, and SJL Mice

**Fig. 1:**
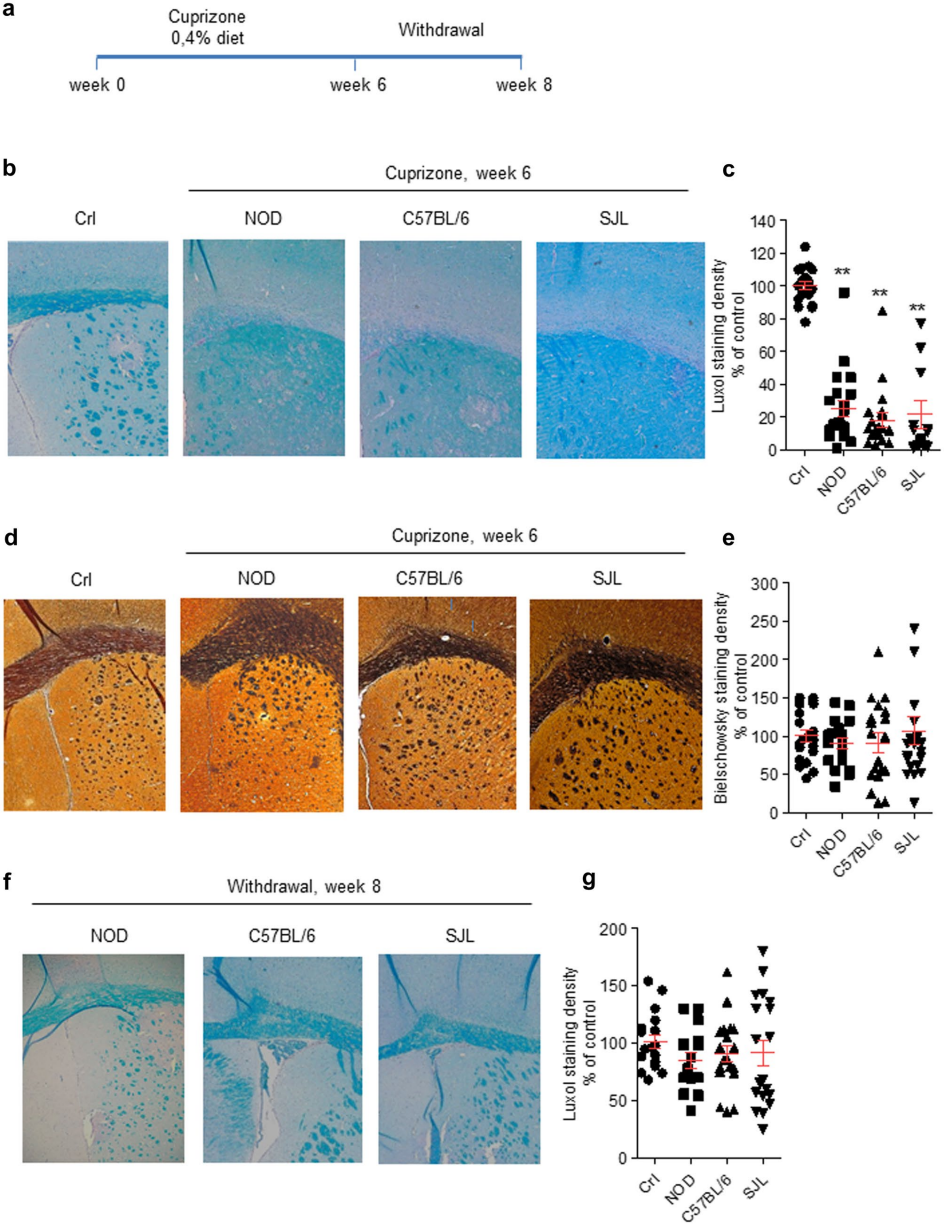
Effects of cuprizone and its withdrawal on demyelination/remyelination of corpus callosum in NOD, C57BL/6 and SJL mice. **a** Time schedule of cuprizone treatment adopted to evaluate demyelination and remyelination in the mouse strains is shown. Luxol Fast Blue **b** or Bielschowsky **d** staining of corpus callosum in control or 6-week cuprizone-exposed mice. A naïve NOD mouse is shown as representative of cuprizone-unexposed mice. **c, e** Quantitation of Luxol Fast Blue or Bielschowsky staining in the callosal area. **f** Luxol Fast Blue staining and its quantitation **g** in the corpus callosum of mice 2 weeks after cuprizone withdrawal. In **c**, **e**, **g**, each column is the mean ± SEM of 5 mice per strain, 4 sections per animal were evaluated (ANOVA plus Tukey’s test). **p < 0.05 vs Crl

Exposure to a cuprizone-containing diet is a prototypical model of demyelination probably due to the copper-chelating effects of the compound in oligodendrocytes (Vega-Riquer et al. 2019). We found that 6 weeks of a cuprizone diet (Fig. 1a) prompted massive demyelination in the corpus callosum of NOD, C57BL/6, and SJL mice. Notably, as revealed by stain densitometry, the extent of demyelination did not differ among the three mouse strains (Fig. 1b and c). To investigate whether myelin loss might be followed by neurodegeneration in some mouse strains, we also evaluated neurofilament integrity in corpus callosum by means of Bielchowsky staining. As shown in Fig. 1d and e, we found no evidence of abnormal neurofilament staining even in those regions undergoing complete demyelination. A prompt remyelination response typically follows cuprizone withdrawal and is sustained by olygodendrocyte precursor cell proliferation. Thus, to compare remyelination efficiency in the three mouse strains, we evaluated callosal myelin content two weeks after having interrupted the exposure to the cuprizone-containing diet. We found that demyelination completely recovered in NOD, C57BL/6, and SJL mice (Fig. 1f and g).

### Effects of Cuprizone on Astrocytes, Microglia, and Oligodendrocyte Progenitor Cells After Cuprizone sExposure in NOD, C57BL/6, and SJL Mice

**Fig. 2:**
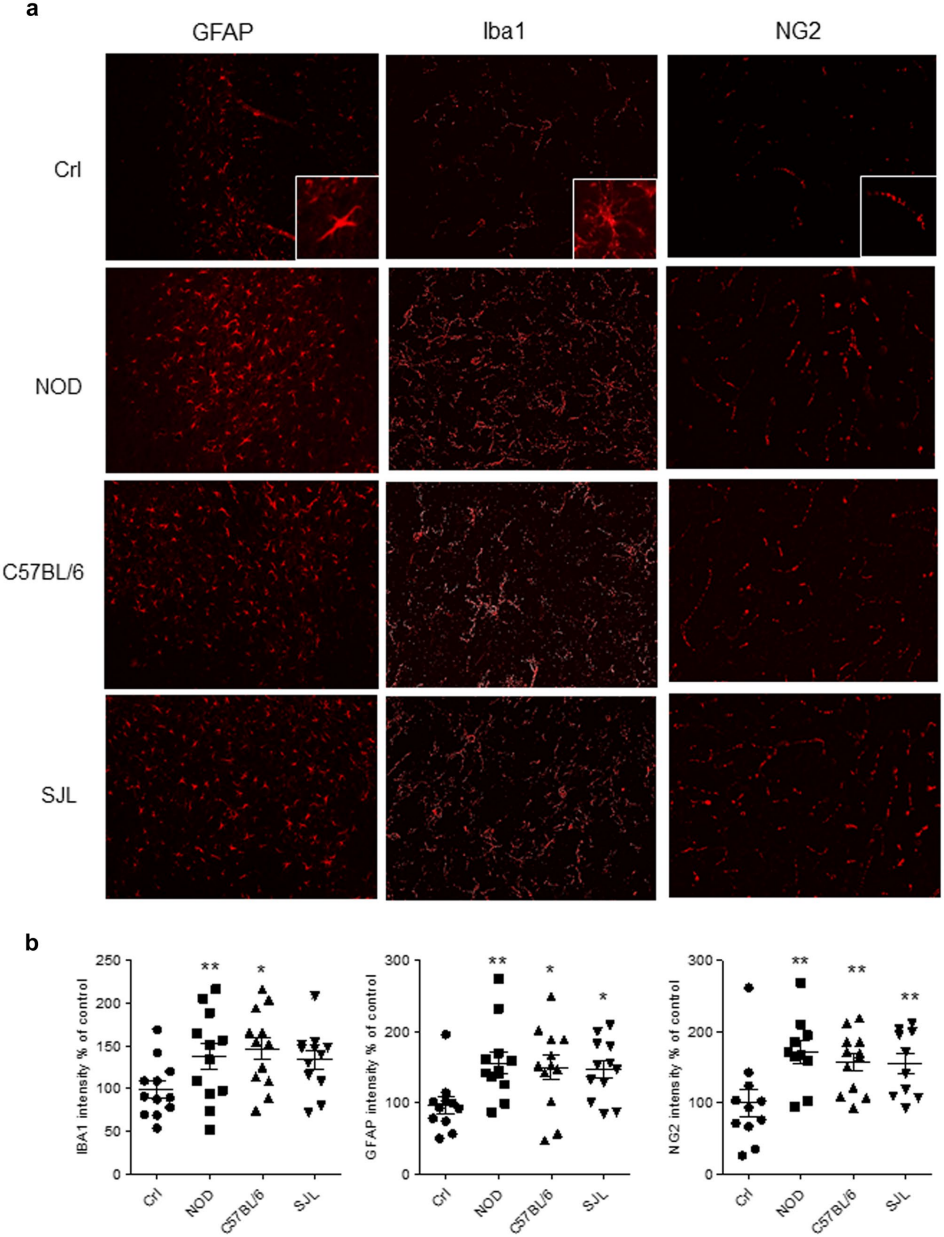
Effects of cuprizone on corpus callosum GFAP, Iba1, and NG2 expression in NOD, C57BL/6, and SJL mice. Representative confocal images **a** and quantitation **b** of immunostaining of astrocytes (GFAP), microglia (Iba1) and oligodendrocyte progenitor cells (NG2) in corpus callosum sections of NOD, C57BL/6, and SJL mice exposed to cuprizone for 6 weeks. In **b**, each column is the mean ± SEM of 4 animals per strain, 3 sections per animal (ANOVA plus Tukey’s test). *p < 0.05, **p < 0.01 vs Crl

Next, we examined whether astrocytes, microglia and OPCs of the 3 mouse strains behaved differently during cuprizone demyelination. We found that 6 weeks after the cuprizone diet immunostaining for GFAP^+^ astrocytes, Iba1^+^ microglia as well as NG2^+^ OPCs dramatically increased in the corpus callosum of the mice, showing however no difference among the strains (Fig. 2).

### Effects of Cuprizone on Functional Parameters During Exposure and Withdrawal From Cuprizone in NOD, C57BL/6, and SJL Mice

**Fig. 3:**
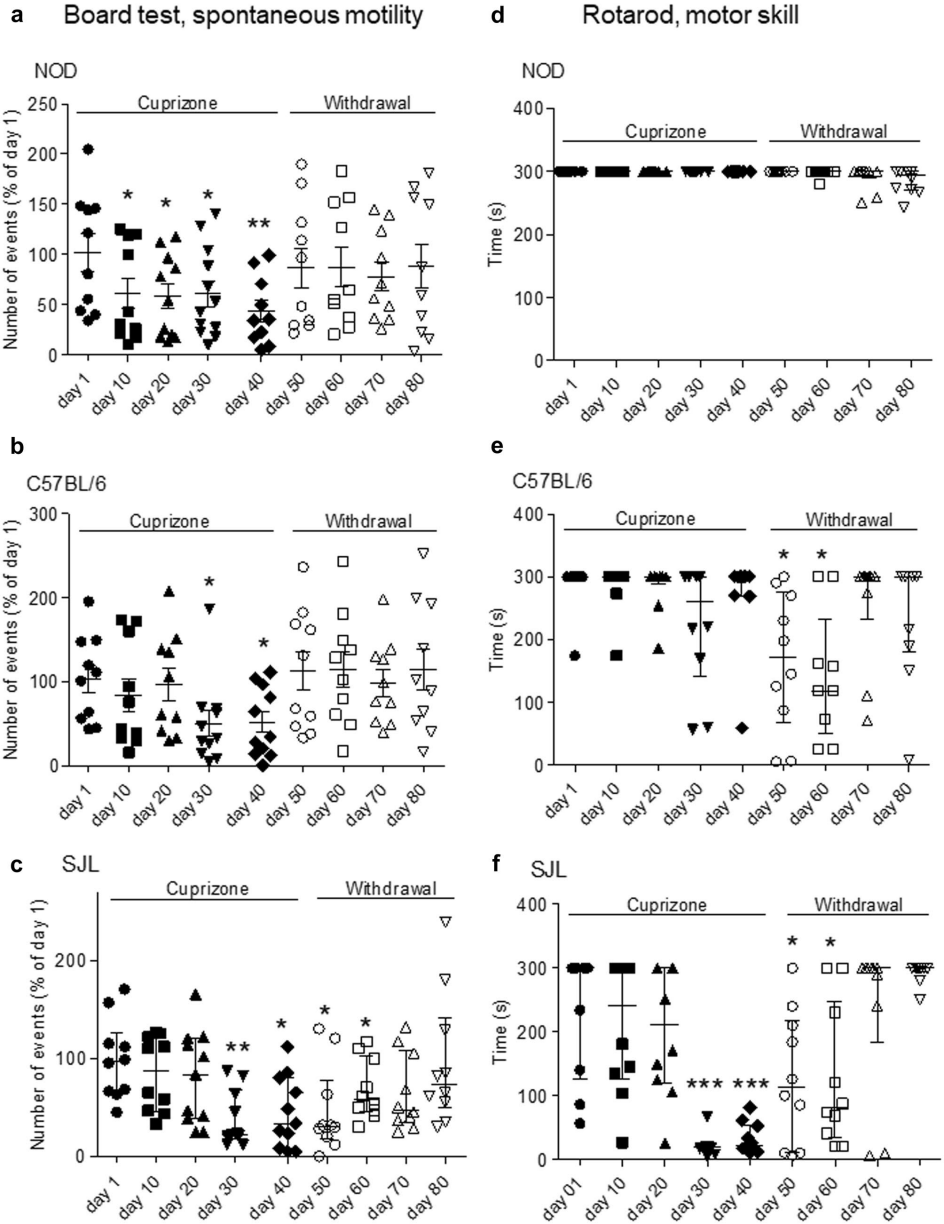
Effects of cuprizone treatment and its withdrawal on neurofunctional parameters in NOD, C57BL/6, and SJL mice. Effects of cuprizone and its withdrawal on spontaneous locomotion **a** and motor skill **b** in NOD, C57BL/6, and SJL mice. In **a** and **b**, each column is the mean ± SEM of 10 mice per strain (ANOVA plus Dunnett’s test). In **c**–**f**, each column is the median and IQR of 10 mice per strain (Kruskal–Wallis and post hoc Dunn’s test). *p < 0.05, **p < 0.01, ***p < 0.001 vs Crl

Because of the apparent lack of difference in cuprizone-dependent demyelination and remyelination responses in the three mouse strains, we then evaluated the neurological deficits during exposure and after withdrawal of the toxicant. We reasoned that, in light of the highly integrated nature of behavioral and motor functions, a neurological examination might better disclose subtle differences of the demyelination/remyelination response among the mouse strains. The board test that evaluates spontaneous motility and the rota road test that evaluates motor skill have been therefore performed every 10 days during the entire period of the experiment (see Experimental Procedures). As far as spontaneous motility is concerned, we found that all of the three mouse strains showed a time-dependent reduction. Interestingly, however, NOD mice showed reduced motility at day 10 after cuprizone exposure, whereas C57BL/6 and SJL mice showed a reduction in spontaneous motility at day 30. All the three mouse strains underwent reductions of motility of the same extent (Fig. 3a–c). Full recovery of spontaneous motility occurred 10 days after cuprizone withdrawal in both NOD and C57BL/6 mice. Conversely, SJL mice showed a much more delayed recovery of motility that reached control values only at day 70 (Fig. 3c). Surprisingly, the rota road test demonstrated that motor skill was not affected in NOD mice throughout the entire course of the experiments (Fig. 3d). C57BL/6 mice also showed unaffected motor skill during cuprizone treatment but, oddly, underwent a delayed and phasic motor skill reduction at day 10 and 20 after cuprizone withdrawal (Fig. 3e). Conversely, SJL mice showed a much more severe impairment of motor skill that began during cuprizone treatment and slowly recovered to control levels (Fig. 3f). Notably, to rule out that motor impairment might be due to diabetes development in NOD mice (Jayasimhan et al. 2014), we measured glycaemia in these animals a weekly basis from week 12 and found that it never exceeded control values (< 250 mg/dL).

## Discussion

For the first time, the present study compared the de/remyelination responses to cuprizone exposure in NOD, C57BL/6, and SJL mice that are typically used to model progressive, chronic, and relapsing–remitting EAE. We show here that callosal demyelination occurs to a similar extent in the three mouse strains and that analogous remyelination kinetics occur upon discontinuation of the cuprizone-containing diet. Even the extent of astrocyte, microglia and OPC activation in the demyelinated area does not differ among the stains. In spite of these analogies, from a functional level the three mouse strains differently respond to the toxicant and its withdrawal. Specifically, NOD mice showed reduction in spontaneous motility faster than that of C57BL/6 and SJL animals, with the latter showing a delayed recovery to control levels. Conversely, motor skill appeared unaffected in NOD mice and transiently reduced in the C57BL/6 and SJL strains. SJL also showed the most severe reduction of motor skill.

Remarkably, the ability of NOD mice to undergo massive callosal demyelination and complete remyelination upon interruption of exposure to the toxicant suggests efficient OPC proliferation. This finding, along with evidence that the extent of the demyelination/remyelination response in NOD mice does not differ with that in SJL animals, that typically undergo 1–3 relapses during EAE but never progress (Cavone et al. 2011, 2014), suggest that EAE progression in NOD animals should not be ascribed to defects of the remyelination response. It is also worth noting that, at variance with NOD mice during EAE (Buonvicino et al. 2019), none of the mouse strains showed sign of neurodegeneration during demyelination. This finding, therefore, provides the first evidence that an intrinsic sensitivity to the demyelinated state is not responsible for axonal degeneration and progression in EAE NOD mice. As a whole, data suggest that neither proneness to demyelination nor impaired remyelination processes underpin disease progression in EAE NOD mice. Rather, the present study, along with data showing an impairment of the T regulatory response in NOD mice (Tang et al. 2008), point to peculiarities of the autoimmune attack during EAE and/or primary proneness to axonopathy in this mouse strain. As for the neuroimmune response, although we recently reported a consistent infiltration of cytotoxic CD8^+^ lymphocytes in the spinal cord of EAE NOD mice during progression, we also noted that reduction of this neuroimmune response by dexamethasone does not provide protection from disease progression (Buonvicino et al. 2019). Apparently, therefore, a key role in determining the peculiar response of NOD mice during EAE is also played by their tendency to undergo neurodegeneration upon inflammation-driven chronic demyelination. It is worth noting, indeed, that activation of innate immunity within demyelinated regions during cuprizone treatment (Fig. 2 and (Skripuletz et al. 2013)) lasts for an extent of time of about 1 month that is shorter than that of 2–4 months necessary for progression onset in NOD animals (Buonvicino et al. 2019). The present findings on the effects of cuprizone in NOD, SJL, and C57BL/6 mice, therefore, add new knowledge on how they respond to a demyelinating challenge, and how efficiently their OPCs promote remyelination. Still, considering that cuprizone-dependent demyelination is massive and acute, we reason that our data do not allow to rule out that a subtle defect in the remyelination response may occur in NOD mice when they are exposed to a less acute, but more prolonged, autoimmune demyelination such as that taking place during EAE. As an additional note, evidence that demyelination is concomitant rather than preceding neurodegeneration in EAE NOD mice (Buonvicino et al. 2019) further suggests that axonopathy is not triggered by defects in the remyelination response.

Data emerging from the comparison of how cuprizone exposure and withdrawal affects neurological functions of the three mouse strains is also of significance. In particular, the earlier reduction of spontaneous motility in NOD mice is in contrast with their complete maintenance of motor skill. Although we do not have an explanation for this apparent inconsistency, it is worth noting that cuprizone-dependent demyelination is massive within the corpus callosum but also occurs within additional brain regions such as the cortex and hippocampus (Koutsoudaki et al. 2009; Skripuletz et al. 2008; Steelman et al. 2012). Possibly, therefore, while spontaneous motility is the results of complex brain networks including both the cortex and hippocampus as well as hemispheric interconnections, motor activity is less dependent from the corpus callosum and more determined by the extrapyramidal and spinal cord network. Accordingly, mice with genetic defects or lesions in the corpus callosum do not show an overt motor impairment (Schalomon and Wahlsten 2002). Interestingly, SJL mice showed a long lasting reduction of spontaneous motility, being able to return to control values only 30 days after cuprizone withdrawal. Recovery of motor skill in SJL mice was more rapid than that of spontaneous motility but showed the highest degree of reduction. These original findings in SJL mice indicate a peculiar sensitivity of the strain to the toxic effects of cuprizone in regions different from the corpus callosum. Data are also in keeping with prior work showing that SJL mice are differently affected by cuprizone compared to those of the C57BL/6 strain (Taylor et al. 2009). Regardless of the difference between SJL and C57BL/6 mice during demyelination, the ability of SJL mice to fully recover motor functions after the several relapses that typically characterize their EAE course tends to rule out a defect in this mouse strain of OPC proliferation and remyelination.

In conclusion, the present study furthers our understanding of the demyelinating–remyelinating response to cuprizone in three mouse strains adopted to model progressive, chronic and RR-EAE. In particular, findings rule out that intrinsic defects of the remyelinating response are causative in determining the progressive nature of EAE in NOD mice, and indicate efficient remyelination in SJL mice in good agreement with their ability to recover from EAE relapses. Data also provide information on the neurofunctional correlates during myelin loss and resynthesis, revealing differences among the strains that may help designing and interpreting future experiments.
